# Environmental Regulation, Resource Misallocation, and Total Factor Productivity: An Empirical Analysis Based on 284 Cities at the Prefecture-Level and Above in China

**DOI:** 10.3390/ijerph20010854

**Published:** 2023-01-02

**Authors:** Xu Dong, Kejia Guo, Guizhi Xue, Yali Yang, Weili Xie, Chenguang Liu

**Affiliations:** 1 School of Economics, Zhengzhou University of Aeronautics, Zhengzhou 450046, China; 2 School of Finance and Taxation, Henan University of Economics and Law, Zhengzhou 450016, China; 3 School of Information Management, Zhengzhou University of Aeronautics, Zhengzhou 450046, China

**Keywords:** total factor productivity, environmental regulation, resource misallocation, resource reallocation effect, city

## Abstract

We investigated the impact of environmental regulation on total factor productivity (TFP) based on a panel dataset of 284 cities at the prefecture-level and above in mainland China from 2006 to 2020 and examined whether environmental regulation had a resource reallocation effect and thus affected TFP. The results showed that there was an “inverted U-shaped” pattern in the impact of environmental regulation on TFP in China and a moderate strengthening of environmental regulation helped to increase TFP, which still held after endogeneity treatment and robustness tests. The “inverted U-shaped” relationship between environmental regulation and TFP in eastern, central, and western cities still held, while environmental regulation did not produce significant effects on TFP in the northeast. The effect of environmental regulation on TFP in large, medium, and small cities tested in groups by city size was consistent with the full sample findings, but the effects decreased in a gradient with city size. The analysis of the impact mechanism showed that environmental regulation had a suppressive effect on resource misallocation and could generate a positive resource reallocation effect and enhance city TFP. The labor reallocation effect of environmental regulation for TFP was stronger than the capital reallocation effect. The findings of our study are of policy reference value for optimizing resource allocation through environmental regulation and thus promoting high-quality city development in China.

## 1. Introduction

Total factor productivity (TFP) measures the contribution of technological progress to economic growth [1], reflects whether economic development is shifting from input-based growth to efficiency-based growth [2], and is an important indicator of the quality of economic development. As the economic development model changes, enhancing TFP has gradually become a very important development demand in the process of policy design and implementation. The 19th CPC National Congress made a major judgment on the general situation of China’s economic development from a brand-new historical perspective, stating that “China’s economy has shifted from a stage of high-speed growth to a stage of high-quality development” and that it is necessary to “promote changes in quality, efficiency and growth drivers in economic development, and improve TFP”. Cities, as independent systems with strong self-organizing functions, are increasingly becoming the core growth pole of a country’s economic development [3]. To a certain extent, improving the TFP of cities is significant for the whole national economy to achieve high-quality development.

According to existing studies in academia, there are two main ways to improve TFP: one is the improvement of production efficiency through technological innovation, and the other is the improvement of allocation efficiency through the optimal reorganization of resources [4]. Technological innovation, while creating high returns, is also accompanied by high risk, and it is difficult to achieve major breakthroughs in the short term, which makes it crucial to seek TFP improvement through optimizing resource allocation. However, due to the insufficiency of market economy development, rigid institutional mechanisms, and local protectionism, the problem of resource misallocation exists to varying degrees in various regions in China and has become an important factor impeding TFP improvement [5].

With the increasingly serious ecological and environmental problems, environmental regulation has become an important policy tool for the government to address environmental pollution and improve the quality of development. As the main spatial carrier for developing industries and promoting urbanization, cities are the most important gathering place for environmental pollution, and investigating the effects of environmental regulation at the city level has received considerable attention from both academia and policy circles. A large number of studies have shown that environmental regulation affects TFP, but the findings are not consistent. A possible important reason is the asymmetry of environmental regulation in the face of heterogeneous spatial units, and this asymmetric rule may produce a resource reallocation effect, which in turn causes uncertainty in the impact on TFP [6,7,8]. In view of this, we must not ignore the possible resource reallocation effect in the process of environmental regulation affecting TFP, and we must investigate all three in a unified analytical framework.

What is the impact of environmental regulation on TFP at the city level in China? Does it have a resource reallocation effect and thus affect TFP? How can environmental regulation policies and resource allocation be optimized to improve TFP? These are important questions worthy of in-depth discussion, and the existing studies leave much to be desired in this regard. Accordingly, we examined the effect of environmental regulation on TFP and the role of resource misallocation in this process with the help of a panel fixed-effect model and a mediating-effect model based on a theoretical analysis of the intrinsic relationship between environmental regulation, resource misallocation, and TFP in a sample of 284 cities at the prefecture-level and above in China.

By reviewing the existing research literature, the possible contributions or innovations of this paper are reflected in the following two aspects. First, we systematically and comprehensively analyzed the theoretical mechanism of environmental regulation affecting TFP and the underlying mechanism of the resource reallocation effect of environmental regulation. Although we discussed this issue in a previous study [4], that study was relatively shallow. In particular, the theoretical explanation of why environmental regulation generates the resource reallocation effect lacked sufficient literature, which we have now enriched and improved. Second, we provide empirical evidence on the TFP growth effect and the resource reallocation effect of environmental regulation at the city level in China. There are only a few papers that investigated the relationship between environmental regulation, resource misallocation, and TFP in one framework, and no city-based empirical findings have been found, so our work can serve to fill this gap in the existing studies.

The rest of this paper is arranged as follows: Section 2 presents a literature review and the corresponding theoretical hypotheses. Section 3 introduces the empirical research design, including model construction, variables, and a description of the data. Section 4 gives the empirical results and discussion, and the final part provides our conclusions and brief policy implications.

## 2. Literature Review and Theoretical Hypotheses

### 2.1. Environmental Regulation and TFP

There are three views regarding the impact of environmental regulation on TFP, which can be summarized as the “constraint hypothesis”, “Porter hypothesis”, and “uncertainty hypothesis”. First, based on the neoclassical framework, the constraint hypothesis argues that environmental regulation causes increases in production costs for firms and a crowding-out effect on productive investment, resulting in TFP loss, and that this constraint effect is gradually transmitted to the industry and regional levels [9,10,11]. Second, the Porter hypothesis states that environmental regulation can stimulate enterprises to strengthen green technological innovation and generate an “innovation compensation” effect that can partially or even fully offset the rising costs caused by environmental regulation, thus achieving an increase in the TFP of enterprises, industries, and even macroeconomies [12,13,14,15]. Third, the “uncertainty hypothesis” suggests that the impact of environmental regulation on TFP is uncertain, and this uncertainty is mainly manifested by the “U-shaped” [16], “inverted U-shaped” [17,18], “inverted N-shaped” [19], “J-shaped” [20], and other nonlinear characteristics between environmental regulation and TFP, i.e., the impact of environmental regulation on TFP is different at different regulatory intensities. From the large number of studies on the relationship between environmental regulations and TFP, the three hypotheses above take firms as the starting point of theoretical analysis and draw different conclusions about the impact of environmental regulation on TFP. Similarly, assuming that a “region (city)” is considered as a specific agent, its TFP is necessarily affected to some extent when it faces environmental regulatory constraints, just like firms. With the above three theoretical hypotheses, we propose the following research hypotheses:

**H1a**  : *Environmental regulation can promote city TFP*.

**H1b**  : *Environmental regulation is not favorable to enhance city TFP*.

**H2**  : *The impact of environmental regulation on city TFP is non-linear as the intensity of regulation rises*.

### 2.2. Resource Misallocation and TFP

There is a basic academic consensus that resource misallocation leads to TFP loss, but the analytical ideas are different. One is known as the “direct approach”, which selects a factor (or several factors) that is theoretically considered to be important and attempts to directly quantify the degree of resource misallocation and TFP loss caused by this factor. Researchers have mainly examined from the perspectives of policy distortions and institutional distortions [21,22,23]. The other idea, called the “indirect approach”, analyzes all potential factors that may lead to resource misallocation and quantifies their impact on TFP by constructing a theoretical model. Generally, researchers examine how the aggregate TFP varies with the resource misallocation coefficient by subsidizing or taxing firms so that they face varying resource misallocation coefficients [24,25]. Hsieh and Klenow (2009) made a pioneering contribution by developing a micro-to-macro theoretical analysis framework based on the degree of TFP dispersion in terms of factor distortions at the firm level and quantifying their impact on the aggregate TFP based on the definition of resource misallocation [26]. Subsequently, a large number of scholars have further refined the theoretical framework along the line that resource misallocation affects TFP [27,28,29]. As for empirical studies, scholars mainly rely on econometric models to estimate the impairment effect caused by resource misallocation on TFP. Among them, most studies take enterprises or industries as the empirical objects [30,31,32], while there is relatively little empirical evidence at the regional level [5]. Based on the above analysis, we propose the following research hypothesis:

**H3:**  *Resource misallocation leads to a significant decrease in city TFP*.

### 2.3. Environmental Regulation and Resource Misallocation

Comparatively speaking, the academic literature devoted to studying the impact of environmental regulation on resource misallocation is rare, and the research methods are mainly based on empirical analysis. The relevant literature is broadly divided into three categories. The first type of literature explores the moderating role of environmental regulation on the impact effect of resource misallocation. Li et al. (2022) examined the moderating role of environmental regulation between land resource misallocation and environmental pollution and argued that the implementation of environmental regulation can suppress the environmental pollution problem caused by land resource misallocation [33]. The second type of literature investigates the mediating role played by resource mismatch in the impact of environmental regulation. The empirical results of Dong et al. (2021) showed that the implementation of environmental regulation policies can increase the TFP of regional industries by reducing the degree of resource misallocation [4]. The third type of literature focuses directly on the effect of environmental regulation on resource misallocation. One view is that environmental regulation distorts resource allocation by increasing production costs and causing some factors of production to flow to sectors or regions with more lenient regulatory policies [34]. Another view points out that the compensatory effect of innovation induced by environmental regulation in the long run will contribute to the efficiency of resource allocation [35,36]. According to the existing literature, it is largely accepted by academia that environmental regulations have a significant resource reallocation effect. Meanwhile, the second and third types of literature mentioned above also suggest that resource misallocation can serve as a transmission path for environmental regulations to affect total factor productivity. Due to the different environmental regulations in different regions, factors of production in regions with higher regulatory intensity tend to flow to regions with lower regulatory intensity due to profit-seeking considerations, but it is uncertain how the resource allocation in the relevant regions changes, which mainly depends on their initial resource allocation status [37]. This means that environmental regulation may either improve resource misallocation and have a positive resource replacement effect on TFP, or it may exacerbate resource misallocation and have a negative resource reallocation effect on TFP. Accordingly, we propose the following research hypotheses:

**H4a**  : *Environmental regulation will help improve resource misallocation in cities and thus enhance their TFP.*

**H4b**  : *Environmental regulation will aggravate resource misallocation in cities and lead to a decrease in their TFP.*

## 3. Empirical Design

### 3.1. Models

First, we investigated the direct impact of environmental regulation on TFP in Chinese cities using a panel two-way fixed effect model to test whether hypotheses H1 and H2 held. The model is specified as follows.

(1) $${\ln\mathrm{TFP}}_{it}{=\alpha }_{0}{+\alpha }_{1}{\ln ER}_{it}{+\alpha }_{2}{\ln ER}_{it}^{2}+\sum \alpha_{j}{Controls}_{jt}{+\mu }_{i}{+\lambda }_{t}{+\varepsilon }_{it}$$

where *i* denotes city and *t* denotes year; *ER* is the environmental regulation intensity, and the squared term of *ER* is used to verify whether there is a non-linear characteristic of the effect of environmental regulation on city TFP. It should be noted that environmental Kuznets theory suggests that the impact of environmental regulation on TFP tends to have only one inflection point if it is non-linear. Therefore, our test of the non-linear impact of environmental regulation only considered its quadratic term. To alleviate the possible heteroskedasticity of the model, the explained variable and explanatory variables were treated logarithmically. *Controls* represents a series of control variables, including the level of economic development, industrial structure upgrading, urban innovation capacity, openness to the outside world, urbanization level, degree of marketization, and government regulation capacity. $\mu_{i}$, $\lambda_{t}$, and $\varepsilon_{it}$ represent the city fixed effect, year fixed effect, and random disturbance terms, respectively.

Second, based on the theoretical analysis, environmental regulation has a resource reallocation effect and may have an impact on city TFP by acting on resource misallocation. We tested this transmission mechanism by using the mediating effect model to verify whether hypotheses H3 and H4 were valid. According to the suggestion of Wen and Ye (2014) [38], the following mediating effect models were constructed:

(2) $${\ln MISK}_{it}{=\beta }_{0}{+\beta }_{1}{\ln ER}_{it}+\sum \beta_{j}{Controls}_{j,\;it}{+\mu }_{i}{+\lambda }_{t}{+\varepsilon }_{it}$$

(3) $${\ln\mathrm{TFP}}_{it}{=\gamma }_{0}{+\gamma }_{1}{\ln MISK}_{it}+\sum \gamma_{j}{Controls}_{j,\;it}{+\mu }_{i}{+\lambda }_{t}{+\varepsilon }_{it}$$

(4) $${\ln\mathrm{TFP}}_{it}{=\delta }_{0}{+\delta }_{1}{\ln ER}_{it}{+\delta }_{2}{\ln MISK}_{it}+\sum \delta_{j}{Controls}_{j,\;it}{+\mu }_{i}{+\lambda }_{t}{+\varepsilon }_{it}$$

(5) $${\ln MISL}_{it}{=\theta }_{0}{+\theta }_{1}{\ln ER}_{it}+\sum \theta_{j}{Controls}_{j,\;it}{+\mu }_{i}{+\lambda }_{t}{+\varepsilon }_{it}$$

(6) $${\ln\mathrm{TFP}}_{it}{=\rho }_{0}{+\rho }_{1}{\ln MISL}_{it}+\sum \rho_{j}{Controls}_{j,\;it}{+\mu }_{i}{+\lambda }_{t}{+\varepsilon }_{it}$$

(7) $${\ln\mathrm{TFP}}_{it}{=\varphi }_{0}{+\varphi }_{1}{\ln ER}_{it}{+\varphi }_{2}{\ln MISL}_{it}+\sum \varphi_{j}{Controls}_{j,\;it}{+\mu }_{i}{+\lambda }_{t}{+\varepsilon }_{it}$$

where *MISK* and *MISL* denote the degree of capital misallocation and the degree of labor misallocation, respectively, and the other variables have the same meanings as above. Equations (2)–(4) were used to test whether environmental regulation affected city TFP through capital misallocation, and Equations (5)–(7) were used to test whether environmental regulation affected city TFP through labor misallocation.

### 3.2. Variables

#### 3.2.1. The Explained Variable

The explanatory variable in this paper is the TFP of each city. Solow residual (SR), data envelopment analysis (DEA), and stochastic frontier analysis (SFA) are the three most widely used TFP measures, among which SR and SFA are parametric estimates and DEA is a non-parametric estimate. Since the parametric estimation method is based on production function and faces relatively strict assumptions, we used the nonparametric DEA-Malmquist index to quantify the TFP values of Chinese cities. The basic principles and steps of this method are as follows:

Suppose there are *N* agents in the sample region. The factor input vector of the *n^th^* agent in period *t* is *x_nt_,* and the output vector is *y_nt_*. *S_t_* denotes the set of production possibilities. Accordingly, the output distance function of the *n^th^* agent in period *t* can be defined as

(8) $$D_{nt}\left(x_{nt}{,y}_{nt}\right)=\inf\left\{\theta :\left(x_{nt},\frac{y_{nt}}{\theta }\right)\in S_{t}\right\}$$

where θ denotes the technical output efficiency. The symbol “inf” is an abbreviation of the word “infimum”, which means the lower bound, because the optimal value of technical output efficiency under the condition of the convex distance function cannot exceed the production frontier. Similarly, the output distance function of the *n^th^* agent in period *t* + 1 is

(9) $$D_{n,t+1}\left(x_{n,t+1}{,y}_{n,t+1}\right)=\inf\left\{\theta :\left(x_{n,t+1},\frac{y_{n,t+1}}{\theta }\right)\in S_{t}\right\}$$

Furthermore, we can define the output distance function $D_{nt}\left(x_{n,t+1}{,y}_{n,t+1}\right)$ for period *t*+1 with reference to the production technology in period *t* and the output distance function $D_{n,t+1}\left(x_{nt}{,y}_{nt}\right)$ for period *t* with reference to the production technology in period *t*+1, i.e.,

(10) $$D_{nt}\left(x_{n,t+1}{,y}_{n,t+1}\right)=\inf\left\{\theta :\left(x_{n,t+1},\frac{y_{n,t+1}}{\theta }\right)\in S_{t}\right\}$$

(11) $$D_{n,t+1}\left(x_{nt}{,y}_{nt}\right)=\inf\left\{\theta :\left(x_{nt},\frac{y_{nt}}{\theta }\right)\in S_{t}\right\}$$

Therefore, the Malmquist indices of the *n^th^* agent with reference to the production technology in period *t* and *t*+1, respectively, are

(12) $$M_{nt}=\frac{D_{nt}\left(x_{n,t+1}{,y}_{n,t+1}\right)}{D_{nt}\left(x_{nt}{,y}_{nt}\right)}$$

(13) $$M_{n,t+1}=\frac{D_{n,t+1}\left(x_{n,t+1}{,y}_{n,t+1}\right)}{D_{n,t+1}\left(x_{nt}{,y}_{nt}\right)}\;$$

For practical studies, the geometric mean of Equations (12) and (13) is usually used as a measure of the TFP index, i.e.,

(14) $$\mathrm{TFPch}=\sqrt{M_{nt}{\times M}_{n,t+1}}$$

Since we are interested in the effect of environmental regulation on the TFP level rather than on its growth rate, we set the TFP of each city to be 1 in the base period and converted the TFP index defined by Equation (14) into the cumulative TFP index to quantify the TFP level of each city [39].

#### 3.2.2. The Core Explanatory Variables

The core explanatory variable in this paper is the intensity of environmental regulation (*ER*) in each city. According to the existing literature, environmental regulation can be divided into formal regulation, which belongs to governmental actions, and informal regulation, which belongs to non-governmental organizations or individual actions [40]. In the current practice of environmental governance in China, the government still plays a dominant role, so the environmental regulation referred to in this paper focuses on the various environmental policies implemented by the government. For the measure of regulation intensity, we used the frequency of environment-related words appearing in each city’s government work report as a proportion of the total word frequency of the full government work report [41]. The key environment-related words included “environmental protection”, “environmental protection” (abbreviated in Chinese context), “pollution”, “energy consumption”, “emission reduction”, “pollution emissions”, “ecology”, “green”, “low-carbon”, “air”, “chemical oxygen demand”, “sulfur dioxide”, “carbon dioxide”, “PM10”, and “PM2.5”.

#### 3.2.3. The Mediating Variables

To clarify the role of resource misallocation between environmental regulation and TFP, the degree of resource misallocations (including capital misallocation and labor misallocation) were treated as the mediating variables. At present, there are two mainstream methods to measure regional resource misallocation in China. One is based on the database of Chinese industrial enterprises and calculates the standard deviation of TFP in each region. The larger the standard deviation, the higher the degree of resource misallocation. The other method is based on the deviation of regional actual factor input from effective factor input. The larger the deviation, the higher the degree of resource misallocation. Although the former is more widely used and the related literature is mostly published in top journals, it is mainly applicable to enterprise- and industry-level studies, and the limitation of timeliness due to the slow updating of the database of Chinese industrial enterprises also seriously restricts the use of this method. In contrast, the second method is more popular for studies at the regional level [42,43]. Therefore, we adopted the second method to measure the degree of resource misallocation in Chinese cities, which is described as follows:

Assume that the production function of each city obeys the Cobb–Douglas form and the scale returns are constant, i.e.,

(15) $$Y_{it}{=A}_{it}K_{it}^{\beta_{Ki}}L_{it}^{\beta_{Li}}$$

Both sides of Equation (15) are taken logarithmically at the same time, and the collation leads to

(16) $$\ln\left(\frac{Y_{it}}{L_{it}}\right){=\ln A}_{it}{+\beta }_{Ki}\ln\left(\frac{K_{it}}{L_{it}}\right){+\mu }_{it}$$

where *Y_it_* is the real GDP of each city, labor input (*L_it_*) is expressed as the number of employees at the end of the year in each city, and capital input (*K_it_*) is expressed as the fixed capital stock in each city. For any city (*i)*, the fixed capital stock is estimated using the perpetual inventory method with the following equation:

(17) $$K_{t}=\frac{I_{t}}{P_{t}}+\left(1-\delta \right)K_{t-1}$$

where *K_t_* and *K_t_*_-1_ denote the current- and previous-period fixed capital stock, respectively. *I_t_* is the current-period fixed asset investment, and *P_t_* is the fixed asset investment price index for the corresponding period. *δ* is the fixed capital depreciation rate, which takes the value of 9.6% [44]. For the base-period fixed capital stock, as suggested by Hall and Jones (1999) [45], the following formula was used:

(18) $$K_{0}=\frac{I_{0}}{\left(\delta +r\right)}$$

where *I*_0_ is the base-period fixed asset investment and *r* is the average annual growth rate of fixed asset investment during the study period.

Due to the differences in economic and technological levels, capital and labor output elasticities may differ across cities, and it is more suitable to use a variable coefficient panel data model for estimation. Specifically, the interaction term between the city dummy variable and the explanatory variables can be introduced in the regression equation, and the coefficient of the interaction term is the capital output elasticity of the corresponding city. Once the capital output elasticity (${\hat{\beta }}_{Ki}$) is found by regression for each city, the labor output elasticity is ${\hat{\beta }}_{Li}{=1-\hat{\beta }}_{Ki}$ due to constant returns to scale. After estimating the capital and labor output elasticities, the absolute factor price distortion coefficients of capital and labor for each city are obtained by substituting the following equations:

(19) $${\hat{\theta }}_{Ki}=\left(\frac{K_{i}}{K}\right)/\left(\frac{s_{i}{\hat{\beta }}_{Ki}}{{\hat{\beta }}_{K}}\right)$$

(20) $${\hat{\theta }}_{Li}=\left(\frac{L_{i}}{L}\right)/\left(\frac{s_{i}{\hat{\beta }}_{Li}}{{\hat{\beta }}_{L}}\right)$$

where $s_{i}$ denotes the share of the output of city *i* in the output of all cities; ${\hat{\beta }}_{K}$ and ${\hat{\beta }}_{L}$ denote the values of capital and labor contributions weighted by $s_{i}$, respectively; and $K_{i}/K$ and $L_{i}/L$ denote the actual proportions of capital and labor used by city *i* in the total capital and labor of all cities, respectively, while $s_{i}{\hat{\beta }}_{Ki}/{\hat{\beta }}_{K}$ and $s_{i}{\hat{\beta }}_{Li}/{\hat{\beta }}_{L}$ are the theoretical proportions of capital and labor used by city *i* when capital and labor are efficiently allocated, respectively.

Furthermore, the degree of capital misallocation and the degree of labor misallocation can be calculated for each city in any given period using the following equations:

(21) $${MISK}_{i}=\left|\frac{1}{{\hat{\theta }}_{Ki}}-1\right|$$

(22) $${MISK}_{i}=\left|\frac{1}{{\hat{\theta }}_{Li}}-1\right|$$

#### 3.2.4. The Control Variables

To weaken the possible estimation bias caused by omitted variables in the models, the following control variables were included:

(1) The level of economic development (ln*rpgdp*), measured as the logarithm of real GDP per capita.
(2) Industrial structure upgrading (*indupgrd*), calculated according to the method provided by Gan et al. (2011) [46].
(3) City innovation capacity (ln*invg*), expressed by adding 1 to the number of invention patents granted and taking the logarithm.
(4) The level of openness to the outside world (*open*), measured by the logarithm of total imports and exports.
(5) The level of urbanization (*urbzn*), measured by the urbanization rate of the resident population, i.e., the proportion of the resident urban population in the total resident population.
(6) The degree of marketization (cmi), calculated according to the method provided by Wang et al. (2021) [47].
(7) Government regulatory capacity (*govrc*), measured as the share of local fiscal expenditure in GDP.

### 3.3. Data

The sample in this paper was a panel of 284 cities at the prefecture-level and above in mainland China from 2006 to 2020. Except for the original data on the number of invention patents granted, which measure the innovation capacity of cities in the control variables and were obtained from the Chinese Research Data Services Platform (CNRDS), the original data required for the measurement of the other variables were obtained from the China City Statistical Yearbook, the statistical yearbooks of the provinces where each city was located, and the statistical yearbooks of the relevant cities. Some variables involving price factors were deflated using 2006 as the base period. Table 1 reports a descriptive statistical overview of the variables of interest used in the models in this study.

## 4. Empirical Results and Discussion

### 4.1. The Benchmark Regression

We first investigated the direct effect of environmental regulation on TFP in Chinese cities using a panel fixed-effect model, and the results are shown in Table 2. Specifically, column (1) reports the estimation results when no control variables were included, and columns (2) to (4) show the estimation results when control variables were included, where columns (2) and (3) control for the city fixed effect and year fixed effect, respectively, while column (4) controls for both. From the estimation results of the models, the two-way fixed-effect model had a better fit, so the next analysis in this paper was based on the two-way fixed-effect model. The estimated coefficient of ln*ER* was significantly positive at the 1% level, indicating that the strengthening of environmental regulation promoted city TFP, which confirms hypothesis H1a and falsifies hypothesis H1b. The estimated coefficient of ln*ER*^2^ was significantly negative at the 5% level, implying an “inverted U-shaped” relationship between environmental regulation and city TFP. This result supports hypothesis H2. Based on the above results, at the city level in China, although environmental regulation is beneficial to enhance TFP, after environmental regulation is strengthened to a certain level, continuing to strengthen it will instead lead to a decline in TFP, which implies that there is an optimal environmental regulation intensity for promoting TFP, and the value of this intensity is about 1.488 (=e^0.089/(2 × 0.112)^).

### 4.2. Endogeneity Treatment

Considering that there is often an inverse causal relationship between environmental regulation and TFP [48], environmental regulation and its squared term in the model may be endogenous, and we intended to adopt an instrumental variable approach to deal with the endogeneity in this paper. Referring to Hering and Poncet (2014) and Qin et al. (2021), the ventilation coefficient was used as an instrumental variable for environmental regulation [49,50]. The ventilation coefficient was the product of the wind speed at a height of 10 m and the height of the atmospheric boundary layer for each city, calculated and compiled from raster data provided by the ERA-Interim database of the European Centre for Medium-Range Weather Forecasts. Column (1) in Table 3 reports the results of the instrumental variable estimation. It can be seen that the K-P (Kleibergen–Paap rk) LM test significantly rejected the null hypothesis. The IV model is not unidentifiable, and the Hansen J test proved that the model could be identified exactly. In addition, both the K-P (Kleibergen–Paap rk Wald) F test and the C-D (Cragg–Donald Wald) F test indicated that the selected instrumental variables did not suffer from a weak correlation problem. The second-stage results of the IV-2SLS show that the coefficients of ln*ER* and ln*ER*^2^ were significant at 1% level and the signs were consistent with the theoretical expectation. Therefore, the relationship between environmental regulation and city TFP was consistent and more significant than the benchmark regression results after mitigating the potential endogeneity problem of the model.

### 4.3. Robustness Tests

To ensure the credibility of the benchmark regression results, we took the following three approaches for robustness testing in this paper: First, we changed the measurement of explanatory variables. Considering the multidimensionality, complexity, and concurrency of environmental governance, a comprehensive environmental regulation intensity indicator (*ERI*) was constructed, as follows, for each city based on industrial wastewater, sulfur dioxide, and smoke (dust) emissions [51]. The model was re-estimated using the new environmental regulation intensity indicator, replacing the original explanatory variables, and the results are shown in column (2) of Table 3.

(23) $${\mathrm{ERI}}_{it}=\frac{{PI}_{it}/{PI}_{t}}{\sum_{s=1}^{3}\left({PE}_{sit}/{PE}_{st}\right)}$$

where *PI_it_* is the industrial pollution control investment of city *i* in year *t* and *PI_t_* is the average of industrial pollution control investment of all cities in year *t*. $\sum_{s=1}^{3}\left({PE}_{sit}/{PE}_{st}\right)$ is the total pollution emission level of *s* kinds of pollution emissions of city *i* in year *t*, in which *PE_sit_* is the *s^th^* pollution emission and *PE_st_* is the average of the *s^th^* pollution emission of all cities in year *t*.

Second, the two-way fixed-effect model was re-estimated by lagging the explanatory variables by one period, which can also attenuate the endogeneity shock to the model to some extent, and the results are shown in column (3) of Table 3. Finally, the model was re-estimated after removing outliers and performing a 5% Winsorization on the sample, and the results are shown in column (4) of Table 3. As can be seen, the direction of the effect of environmental regulation on city TFP remained consistent with the benchmark regression under the three tests, except for slight changes in the absolute magnitude and significance of the regression coefficients, indicating that the previous results were quite robust.

### 4.4. Heterogeneity Analysis

The benchmark regression results initially validated the theoretical hypotheses of this paper on the relationship between environmental regulations and city TFP, but whether the results differed across cities needed to be further explored.

First, the heterogeneity test was conducted from the perspective of city location distribution to examine the variability of the impact of environmental regulation on TFP in the eastern, central, western, and northeastern regions, and the results are shown in Table 4. It can be seen that the coefficient of ln*ER* was significantly positive and the coefficient of ln*ER*^2^ was significantly negative in eastern, central, and western cities, indicating that the “inverted U-shaped” impact of environmental regulation on TFP still held in these regions. In Northeast China, the coefficients of ln*ER* and ln*ER*^2^ did not pass the significance test, although the direction was consistent with the benchmark regression, implying that environmental regulation has not yet had a significant effect on city TFP in Northeast China. The possible reason for this may be that Northeast China has suffered from a lack of economic development momentum and efficiency decline due to population loss and slow transformation in recent years, and the positive effects of environmental regulation are difficult to be seen in the short term compared with investment, consumption, and exports, which are more obvious means to promote economic development.

Second, the heterogeneity test was conducted from the perspective of city size distribution to examine the variability of the impact of environmental regulation on TFP among large, medium, and small cities, and the results are shown in Table 5. It can be seen that the effects of environmental regulation on TFP in large, medium, and small cities were fully consistent with the results of the benchmark regression, with the largest boosting effect of environmental regulation on TFP in large cities, followed by medium and small cities. The result also implies that the pattern of the benign development of large, medium, and small cities in China was naturally formed, and environmental regulation is becoming a powerful policy tool for cities of different sizes to promote the efficiency of economic development.

### 4.5. Impact Mechanism Examination

The previous theoretical analysis suggested that environmental regulation may act on city TFP by influencing resource misallocation to produce a resource reallocation effect, and a mediating effect model was employed to test whether this transmission mechanism held. The results are shown in Table 6. From columns (1) to (3), we can find that the effect of environmental regulation on capital misallocation was negative and passed the significance test at the 5% level and that the effect of capital misallocation on TFP was significantly negative at the 1% level, while environmental regulation was still significantly positively related to TFP, which means that environmental regulation can improve capital misallocation and thus increase TFP in Chinese cities. From columns (4) to (6), the regression coefficient of labor misallocation on environmental regulation was significantly negative at the 1% level, and the effect of environmental regulation on TFP was significantly positive, while the effect of labor misallocation was significantly negative, which means that strengthening environmental regulation can improve labor misallocation and thus promote TFP in Chinese cities. Comparing the estimated coefficients of lnER in columns (1) and (4), environmental regulation is more effective in improving labor misallocation, resulting in lower total factor productivity loss due to labor misallocation than capital misallocation. The mechanism test results confirm theoretical hypotheses H3 and H4a, indicating that environmental regulation has a positive resource reallocation effect and can promote TFP in Chinese cities by suppressing resource misallocation.

## 5. Conclusions

Based on panel data of 284 cities at the prefecture-level and above in China from 2006 to 2020, this paper empirically investigated the impact of environmental regulation on TFP and the mediating mechanism played by resource misallocation in the process using a panel two-way fixed-effect model and a mediating effect model. The main findings are as follows:

(1) Environmental regulation had a direct boosting effect on TFP in Chinese cities, but the intensity of regulation was not as high as it should be. The relationship between environmental regulation and TFP showed an “inverted U-shaped” characteristic, and over-strengthening environmental regulation was not conducive to TFP improvement. The results still held after endogeneity treatment and robustness tests. For our sample data, the average optimal environmental regulation intensity of Chinese cities was about 1.488.
(2) The impact of environmental regulations on TFP in Chinese cities was heterogeneous in terms of location and scale. In terms of location distribution, the impact of environmental regulations on the TFP values of cities in the eastern, central, and western regions maintained the “inverted U-shaped” trend of “promoting first and then inhibiting”, while it did not have a significant impact on cities in the northeastern region. In terms of city size distribution, the impact of environmental regulation on the TFP values in large, medium, and small cities had the same results as the benchmark regression, i.e., the conclusion of an “inverted U-shape” still held, while the TFP-boosting effect of environmental regulation showed a pattern of “large cities > medium cities > small cities”.
(3) Environmental regulation produced a positive resource reallocation effect by suppressing resource misallocation, and this effect further promoted city TFP. In terms of factor resource types, environmental regulation promoted TFP by improving labor misallocation more effectively than the improvement effect on capital misallocation.

The above research findings suggest the following policy implications:

First, we should adhere to green development and improve the targeting of environmental regulation policies. On the one hand, all cities should consider the actual local economic development and environmental governance, optimize the environmental regulation pattern, and design an appropriate intensity of environmental regulation so that it can fully play its role in promoting total factor productivity. On the other hand, local governments should strengthen the coordination of different environmental regulation tools and avoid adopting “one-size-fits-all” policies; meanwhile, it is necessary to guide the integration and matching of environmental regulation tools with fiscal, financial, and land policies and establish a sound incentive mechanism for environmental protection.

Second, we should adhere to the goal-oriented approach and promote environmental regulation to play a positive resource reallocation effect. On the one hand, the reform process of capital and labor markets should be accelerated, a market-oriented allocation of finance (credit) should be promoted, the resident population registration system should be improved, the household registration barriers to the free mobility of labor should be broken down, and total factor productivity losses caused by resource mismatch should be mitigated. On the other hand, we should continue to adhere to environmental policy, moderately strengthen the intensity of environmental regulation, make pollution control a constraint in the assessment of local governments, mobilize the initiative and enthusiasm of them to optimize resource allocation by environmental means, and further weaken the negative impact of resource misallocation on TFP.

Third, we should adhere to the philosophy of regional coordination and promote synergistic innovation in environmental governance in different cities. The heterogeneity analysis showed that there were differences in the TFP-promoting effect of environmental regulation in different cities. In the context of vigorously promoting coordinated regional development and common prosperity in China, it is also worthwhile to pay attention to how environmental regulation policies promote TFP in different cities toward equilibrium. We suggest that cities of different regions and scales should widely carry out coordinated cooperation and innovation in the field of environmental governance, realize the spatial linkage of regulatory policies, build an open sharing platform for resources, and maximize the spillover effect of environmental collaborative innovation on TFP.

## Figures and Tables

**Table 1:** Descriptive statistics of the variables.

Variable	Obs.	Mean	S.d.	Median	Min	Max
lnTFP	4260	0.641	0.085	0.663	0.128	1.014
ln*ER*	4260	0.276	0.104	0.268	0.018	0.806
ln*ER*^2^	4260	0.087	0.066	0.072	0.000	0.650
ln*MISK*	4260	0.257	0.192	0.225	0.000	1.390
ln*MISL*	4260	0.339	0.244	0.323	0.000	2.006
ln*rpgdp*	4260	10.299	0.725	10.297	7.730	12.416
*indupgrd*	4260	0.964	0.544	0.839	0.089	5.350
ln*invg*	4260	4.362	1.940	4.190	0.000	11.053
*open*	4260	11.795	2.151	11.741	0.693	17.800
*urbzn*	4260	51.695	16.771	49.995	0.000	118.840
*cmi*	4260	10.639	2.799	10.606	3.037	19.694
*govrc*	4260	18.622	10.439	15.928	2.442	148.516

**Table 2:** Environmental regulation and city TFP: the benchmark regression.

Variable	lnTFP			
	(1)	(2)	(3)	(4)
ln*ER*	0.140 ***	0.171 ***	0.127 ***	0.089 ***
	(3.428)	(4.675)	(3.542)	(2.674)
ln*ER*^2^	−0.149 **	−0.204 ***	−0.147 ***	−0.112 **
	(−2.529)	(−3.769)	(−2.870)	(−2.360)
*Controls*	No	Yes	Yes	Yes
Cluster City	Yes	Yes	Yes	Yes
City FE	Yes	Yes	No	Yes
Year FE	Yes	No	Yes	Yes
*R* ^2^	0.592	0.607	0.588	0.653
*N*	4260	4260	4260	4260

Notes: The t-statistics are in parentheses; ** *p* < 0.05, *** *p* < 0.01.

**Table 3:** Environmental regulation and city TFP: endogeneity treatment and robustness tests.

Variable	(1)	(2)	(3)	(4)
	lnTFP (IV-2SLS, 2nd Stage)	lnTFP (New Explanatory Variables)	L.lnTFP	lnTFP (Winsorization)
ln*ER*	0.198 ***		0.045 **	0.141 ***
	(10.185)		(2.280)	(3.343)
ln*ER*^2^	−0.212 ***		−0.023 **	−0.180 ***
	(−14.852)		(−2.452)	(−2.630)
ln*ERI*		0.035 **		
		(2.537)		
ln*ERI*^2^		−0.006 *		
		(−1.778)		
*Controls*	Yes	Yes	Yes	Yes
Cluster City	Yes	Yes	Yes	Yes
City FE	Yes	Yes	Yes	Yes
Year FE	Yes	Yes	Yes	Yes
Hansen J test	47.561 *** [0.000]			
K-P LM test	41.398 *** [0.000]			
K-P F test	14.196 * {7.03}			
C-D F test	9.136 * {7.03}			
*R* ^2^	0.286	0.655	0.655	0.667
*N*	4260	4260	3976	4260

Notes: The *t*-statistics are in parentheses; the *p*-values of related tests are in square brackets; the critical value of the Stock–Yogo weak ID test for IVs at the 10% level is in braces; * *p* < 0.1, ** *p* < 0.05; *** *p* < 0.01.

**Table 4:** Environmental regulation and city TFP: heterogeneity tests (1).

Variable	lnTFP			
	(1) Eastern Cities	(2) Central Cities	(3) Western Cities	(4) Northeastern Cities
ln*ER*	0.224 ***	0.170 **	0.261 ***	0.148
	(3.490)	(2.056)	(3.118)	(1.184)
ln*ER*^2^	−0.313 ***	−0.253 **	−0.375 ***	−0.307
	(−3.071)	(−2.476)	(−2.663)	(−1.331)
*Controls*	Yes	Yes	Yes	Yes
Cluster City	Yes	Yes	Yes	Yes
City FE	Yes	Yes	Yes	Yes
Year FE	Yes	Yes	Yes	Yes
*R* ^2^	0.481	0.758	0.670	0.664
*N*	1290	1200	1260	510

Notes: The t-statistics are in parentheses; ** *p* < 0.05, *** *p* < 0.01.

**Table 5:** Environmental regulation and city TFP: heterogeneity tests (2).

Variable	lnTFP		
	(1) Large Cities	(2) Medium Cities	(3) Small Cities
ln*ER*	0.275 ***	0.253 ***	0.252 ***
	(3.466)	(4.188)	(3.084)
ln*ER*^2^	−0.292 ***	−0.396 ***	−0.323 **
	(−3.070)	(−3.991)	(−2.382)
*Controls*	Yes	Yes	Yes
Cluster City	Yes	Yes	Yes
City FE	Yes	Yes	Yes
Year FE	Yes	Yes	Yes
*R* ^2^	0.561	0.685	0.689
*N*	1290	1290	1680

Notes: The *t*-statistics are in parentheses; ** *p* < 0.05, *** *p* < 0.01.

**Table 6:** Mechanism of environmental regulation affecting city TFP: Resource reallocation effect.

Variable	(1)	(2)	(3)	(4)	(5)	(6)
	ln*MISK*	lnTFP	lnTFP	ln*MISL*	lnTFP	lnTFP
ln*ER*	−0.034 **		0.056 ***	−0.198 ***		0.056 ***
	(−2.030)		(7.732)	(−4.821)		(7.558)
ln*MISK*		−0.026 ***	−0.018 ***			
		(−2.598)	(−5.322)			
ln*MISL*					−0.021 ***	−0.008 ***
					(−2.625)	(−2.983)
*Controls*	Yes	Yes	Yes	Yes	Yes	Yes
Cluster City	Yes	Yes	Yes	Yes	Yes	Yes
City FE	Yes	Yes	Yes	Yes	Yes	Yes
Year FE	Yes	Yes	Yes	Yes	Yes	Yes
*R* ^2^	0.082	0.646	0.648	0.112	0.646	0.647
*N*	4260	4260	4260	4260	4260	4260

Notes: The *t*-statistics are in parentheses; ** *p* < 0.05, *** *p* < 0.01.

## Data Availability

The raw datasets used in this paper can be obtained by contacting the corresponding author.
